# Limb Salvage and Pelvic Reconstruction With Endoprosthesis After Pelvic Tumor Resection: A Narrative Review

**DOI:** 10.7759/cureus.56043

**Published:** 2024-03-12

**Authors:** Anastasios G Roustemis, Markos Liontos, Ioannis Trikoupis, Vasileios Karampikas, Stavros Goumenos, Panagiotis Gavriil, Vasileios A Kontogeorgakos, Olga Savvidou, Panayiotis J Papagelopoulos

**Affiliations:** 1 First Department of Orthopedic Surgery, National and Kapodistrian University of Athens, Athens, GRC; 2 Medical School, Attikon University General Hospital, Athens, GRC; 3 Department of Orthopedic Surgery, Center for Musculoskeletal Surgery, Virchow Klinikum, Charité - Universitätsmedizin Berlin, Berlin, DEU

**Keywords:** pelvic reconstruction, lumic, periacetabular tumors, sarcoma, endoprosthetic reconstruction

## Abstract

Periacetabular defects following tumor resection present formidable challenges in reconstruction and continue to pose clinical difficulties. Historically, treatment approaches leaned towards hindquarter amputation; however, due to associated morbidities and functional limitations, limb-sparing procedures gained prominence in the 1980s. Nevertheless, the intricacies of pelvic anatomy and the imperative of achieving wide surgical margins while preserving essential structures make pelvic tumor resection and subsequent reconstruction inherently complex. Various reconstruction modalities have been explored, including non-vascularized fibular grafts and prosthetic implants.

Among these options, the LUMiC® endoprosthesis stands out as a promising solution for pelvic reconstruction post-tumor resection. Characterized by a modular design featuring a hydroxyapatite-coated stem and acetabular cup, this device has shown favorable implant survival rates in studies, despite encountering complications primarily associated with soft tissue failure, dislocation, and infection. Notably, the incidence of complications varies across studies. The Henderson classification system delineates these complications, encompassing soft tissue issues, aseptic loosening, periprosthetic fractures, infections, and tumor recurrence.

Despite the encouraging functional outcomes associated with the LUMiC® endoprosthesis, it is not immune to limitations. Concerns persist regarding complications such as dislocation and infection, underscoring the imperative for further research to evaluate the long-term durability and reliability of this reconstructive approach. Moreover, advancements in surgical techniques, perioperative management, and the advent of navigation-assisted procedures hold promise for enhancing outcomes and mitigating complication rates in pelvic reconstruction surgeries.

## Introduction and background

Primary bone and soft tissue tumors in the pelvic region are rarely encountered malignancies that make up roughly 14% of all primary bone sarcomas. Among these tumors, chondrosarcoma is the most prevalent tumor type in adults, while osteosarcoma and Ewing's sarcoma are more common in children. Additionally, destructive tumors like giant cell tumors can also affect the pelvis [1]. In the past, hindquarter amputation was the standard treatment for these tumors, but it was associated with significant morbidity and major cosmetic and functional impairments [2].

During the 1980s, there was a shift towards limb salvage procedures, categorized by Enneking and Dunham [3]. Limb salvage surgery in cases of pelvic tumors is challenging due to the complex pelvic anatomy, often distorted by the tumor. Achieving a negative surgical margin to prevent local recurrence is difficult and sometimes impossible due to the close proximity of the tumor to vital structures. Variable treatment methods, including excision arthroplasty, have been used to preserve some hip function, but they can result in pain and leg length discrepancy [4].

Advancements in pre-operative imaging, chemotherapy, and peri-operative care as well as new implants have made it possible to reconstruct the pelvis, aiming for better functionality, psychosocial well-being, and cosmetic outcomes [5]. Non-vascularized fibular grafts have shown positive results, but complications like graft failure, nonunion, fractures, and infections can be severe [6]. Recent studies have focused on endoprosthetic reconstruction of the pelvis after tumor resection. While custom-made endoprostheses have shown acceptable long-term results, they are associated with a high risk of severe complications such as infection and dislocation [7].

Several surgical techniques have emerged for reconstructing the periacetabular region post-tumor resection, including endoprosthetic replacement, allograft and autograft reconstruction, arthrodesis involving the iliofemoral or ischiofemoral joints, and hip transposition [8,9]. The results of reconstructive procedures employing a prosthesis featuring stemmed acetabular components were initially documented by the Birmingham group in 2003. During this period, a novel pelvic replacement technique, referred to as the "ice cream" cone prosthesis, was introduced. This innovation aimed to streamline the reconstruction process, minimize complications, and provide adaptability, particularly in cases with limited residual pelvic structure [10]. Another noteworthy development is the LUMiC prosthesis, a modular implant originating from Germany. This prosthesis is characterized by a distinct design, incorporating a hydroxyapatite (HA)-coated stem and acetabular cup, contributing to its modular functionality [11].

The purpose of this review is to summarize the studies where the LUMiC® endoprosthesis was implemented for pelvic reconstruction after limb salvage procedures.

## Review

Reconstruction survivorship

Implant durability is a critical concern during the selection of the most adequate reconstruction technique. Prosthetic implants must be stable enough to withstand mechanical stress and remain intact over an extended period. In a study conducted by Bus et al., of the 47 patients who underwent pelvic reconstruction following tumor resection, implant failure rates due to mechanical reasons at two and five years were 2.1% and 17.3%, respectively [11]. Guzik included in his study six cases of pelvic reconstruction after hemipelvectomy type 2 with implantation of the LUMiC® endoprosthesis and reported no implant failure during follow-up [12].

In another study by Erol and colleagues, 21 patients with primary bone sarcomas and metastatic lesions of the pelvis were treated with internal hemipelvectomy and endoprosthetic reconstruction of the periacetabular defect with LUMiC® prosthesis. In a median follow-up of 57.8 months, implant survival rates at one, two, and five years were 95.2%, 85.7%, and 80.9%, respectively [13].

Variable reoperation rates following pelvic reconstruction and reconstruction with LUMiC® endoprosthesis have been demonstrated. Guzik reported no reoperations, while in the study by Erol et al., a reoperation rate of 28.5% was reported. Bus et al. reported that 25 of the 47 patients who underwent surgery required reoperation, with the majority of these patients requiring multiple surgeries [11,12]. Recently, Rizkallah and colleagues reported their results of 16 patients with LUMiC® endoprosthesis who were surgically managed with pelvic tumor resection and reconstruction in five Orthopedic Oncology Canadian centers. The study follow-up was 28 months (range: 3-60 months), and during this period, the highest reoperation rate was reported, compared to other studies, with a reoperation rate of 62.5% occurring within the first two years [14].

Another study reports 8.8% (95% CI 1.7-20.1 two-year cumulative incidence of failure), while the four-year cumulative incidence of failure was 14.1% (95% CI 2.2-33.1) [15].

**Figure 1:**
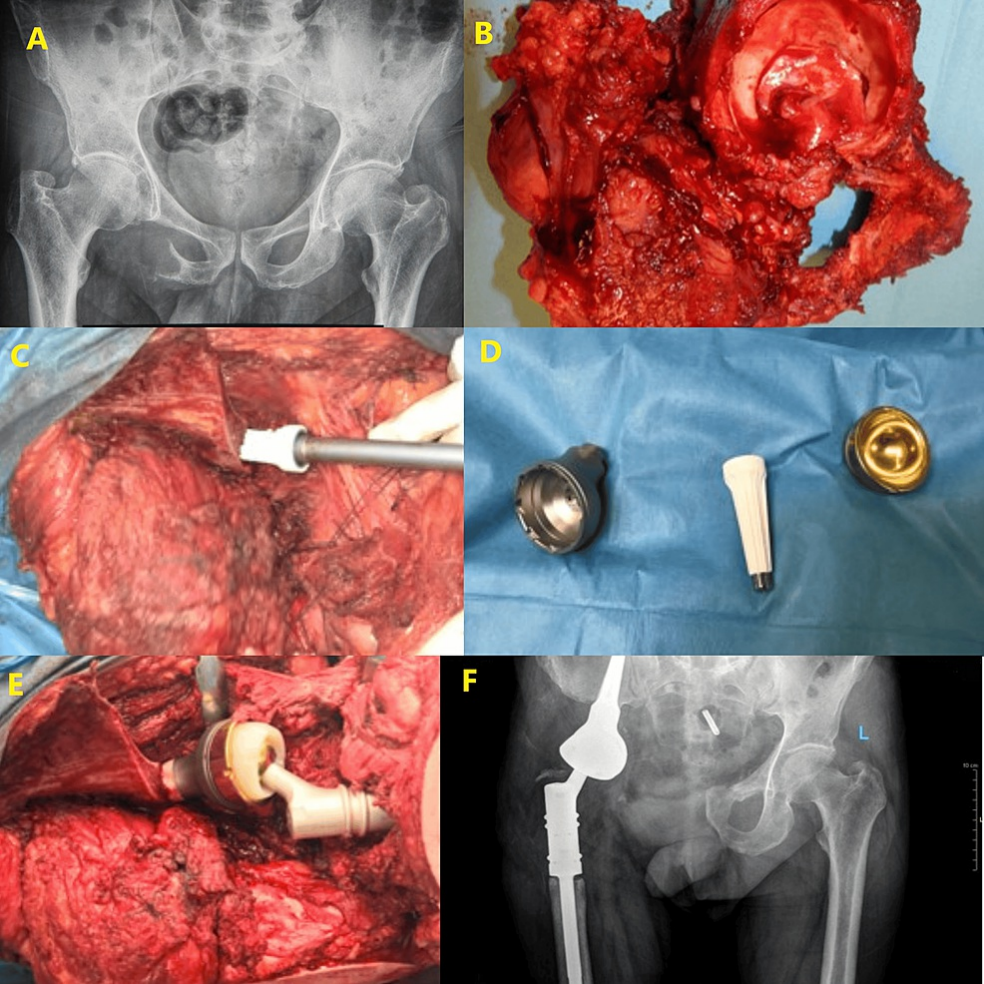
A: The pre-operative radiograph of a 50‐year‐old male patient with chondrosarcoma of the pelvis. B: The patient underwent a wide resection. C: Bone preparation. D: LUMiC endoprosthesis. E: A proximal femur replacement with a LUMiC endoprosthetic device and trevira tube were placed. F: One-year follow-up with radiograph.

Below are images depicting a 50-year-old patient undergoing treatment for chondrosarcoma using a LUMiC device in our university department (Figure 1).

Classification of complications

The utilization of the LUMiC® pelvic reconstruction system aims to establish a stable structure that enables early weight-bearing and delivers satisfactory results with a brief recovery period [13]. While an endoprosthetic replacement has gained popularity for managing periacetabular lesions, there are inherent challenges associated with endoprosthetic reconstruction and concerns related to the patient's oncological care. Reconstructive failures are frequent in these cases, especially since many of these patients are debilitated due to their oncological condition, malnourishment, and adjunctive therapies like chemotherapy, leading to high complication rates [16].

Henderson and colleagues introduced a failure mode classification system widely adopted to categorize the modes, frequency, and timing of implant failures in patients treated with endoprostheses after limb salvage surgeries. In their multicenter, retrospective review, they identified five primary failure modes: implant revision, periprosthetic fracture requiring fixation, soft tissue failure, implant removal without revision, and amputation. These failures were further divided into mechanical and nonmechanical types of failures [17].

Type 1: Soft Tissue Complications

Limb preservation in cases of pelvic sarcomas involving the acetabulum and pelvis represents a formidable surgical challenge for orthopedic oncologists specializing in musculoskeletal conditions. In the study conducted by Bus and colleagues, it was demonstrated that a solitary dislocation occurred in six patients, constituting 13% of the cases. Among these cases, four patients experienced recurrent dislocations, accounting for 9% of the total cases. Notably, one of these patients suffered a primary dislocation following the resection of an extensive tumor recurrence [11].

The initial dislocation occurred within a median timeframe of 20 days, ranging from one day to 2.6 months post-operation. Patients experiencing a single dislocation were managed through either an open (in three cases) or a closed (in three cases) reduction approach. In contrast, two patients encountering recurrent dislocations underwent revision surgery involving the implementation of a dual-mobility cup, yielding favorable outcomes with no subsequent dislocations. Other cases necessitated open reduction and reinforcement using an attachment tube [11].

It is worth noting that Guzik did not observe any type 1 complications throughout the duration of their study. Additionally, Weisstein identified a 9% recurrence rate for dislocations, affecting four out of 47 patients during the study period [18]. Erol et al. reported that soft tissue failure of type 1, resulting in dislocation, was documented in two patients, representing 9.5% of cases. Importantly, both of these occurrences were diagnosed within the first two weeks post-operation [13].

Type 2: Aseptic Loosening

According to Bus et al., aseptic loosening, specifically categorized as Henderson type 2, was observed in three reconstructions with LUMiC® endoprosthesis, accounting for 6% of the cases. Among these cases, two experienced loosening when an uncemented stem was used. In one case, this loosening occurred 57 months after the initial fixation with a structural pelvic allograft, which had failed due to allograft resorption. In another case, which happened 36 months after implantation, an intraoperative fracture led to inadequate primary fixation. The third instance of loosening occurred when a cemented stem was employed [11]. Again, Guzik did not observe any type 2 complications and so did the multicenter Canadian study by Rizkallah and colleagues [12,14]. Erol et al. reported that only one patient required revision of the LUMiC® prosthesis at 18 months post-operatively due to implant loosening [13].

Type 3: Periprosthetic Fracture or Structural Breakage

Similar to type 2 failures noted in reconstruction with the ice cone prostheses, high bending forces at the interfaces between the prosthesis and bone, as well as between the proximal body and stem, along with potential compromise in bone quality among oncology patients increase the susceptibility to periprosthetic or implant fractures. The reported rates of these type 3 failures varied from 0% to 9% over an average follow-up period ranging from 0.25 to 5 years [11,13,14,18].

Type 4: Infection

The most frequently encountered complication in pelvic reconstruction after tumor excision is post-operative periprosthetic joint infection, with varying infection rates reported in different studies, ranging from 0% to 50% [11,13,14,18]. Infection can have significant financial and physical consequences for patients, as it often necessitates revision and prolonged antibiotic administration. In a retrospective study conducted by Rizkallah and colleagues, including 16 patients who underwent pelvic endoprosthetic reconstruction with LUMiC® endoprosthesis, 50% of the cohort encountered post-operative infection involving the prosthesis. This complication emerged as the predominant complication within this specific case series [14]. The high risk for infection is thought to arise from a confluence of patient-related variables and the nature of the surgical intervention [19]. Chemotherapy has been demonstrated to elevate the overall revision rate of prosthetic reconstruction by 30%, due to diminished osseointegration of the implants [20]. Nevertheless, it remains uncertain whether chemotherapy influences the infection rate subsequent to prosthetic reconstruction and whether chemotherapy-induced immunodeficiency constitutes a risk factor for infection [21]. Moreover, radiation therapy can render the surgical incision more vulnerable to breakdown, fostering subsequent infection. Procedure-related factors contributing to the risk of infection encompass extensive dissections, resulting in prolonged exposure times of the incision and the formation of necrotic spaces within soft tissues [22]. Among the metals recognized for their antimicrobial properties, silver has captured the attention of numerous researchers owing to its remarkable antimicrobial efficacy and minimal toxicity [23,24]. In a study including patients with sarcomas, Hardes and colleagues discovered that silver-coated megaprostheses had a significantly lower infection rate when compared to titanium implants [25].

Type 5: Tumor Recurrence

In several cases, the failure of pelvic reconstruction with an endoprosthesis can be attributed to the local recurrence of the tumor. Previous research has indicated that this form of failure is more prevalent when the prosthesis is implemented for the reconstruction after resection of a primary tumor rather than to metastatic bone disease within the pelvis. Apparently, patients with metastatic disease might have a higher likelihood of early mortality, causing the implant to outlast patient survival [26]. There is no consensus concerning the prevalence of complications due to tumor recurrence. Fujiwara et al. documented a complication rate of 23% due to local recurrence, in contrast to Fisher et al., who presented a lower complication rate of 7.4%. Both studies reported their outcomes after pelvic reconstruction with the ice cream cone prosthesis after resection of pelvic tumors [10,27]. Bus et al. reported a local recurrence rate of 13% following the reconstruction with LUMiC® endoprosthesis after limb salvage surgery [11]. Typically, the surgical approach to address this type of failure involves amputating the affected extremity rather than attempting further revision procedures. In contrast, supportive adjuvant therapy is more commonly employed for systemic disease in these cases [28] (Table 1).

**Table 1:** Reported implant survival and complication rates of included studies.

Study	Patients (n)	Follow-up (months)	Implant survival	Type 1 (%)	Type 2 (%)	Type 3 (%)	Type 4 (%)	Type 5 (%)	Reoperation (%)
Bus et al. [11]	47	24	2.1% and 17.3% implant failure for mechanical reasons and 6.4% and 9.2% for infection at two and five years	13	6	9	28	13	53
Guzik [12]	6	12	100%	0	0	0	0	0	0
Erol et al. [13]	21	57.8	95.2% and 80.9% at one and five years	9.5	4.7	0	9.5	9.5	28.5
Rizkallah et al. [14]	16	28 (range: 3-60)	-	5	0	0	8	0	62.5

## Conclusions

Pelvic reconstruction following tumor resection is a critical component of orthopedic oncology, with significant implications for patient quality of life and functional outcomes. Despite the challenges associated with implant durability and complications, advancements in surgical techniques and implant designs offer promising avenues for improving patient care. 

In conclusion, the LUMiC® endoprosthesis can be a useful surgical technique for the reconstruction of the pelvis following tumor resection or failed prior reconstruction methods, as it seems that can lead to satisfying functional results. However, it is associated with several severe complications such as dislocation and infection. Additional research with longer follow-up periods and larger cohorts is imperative to examine the durability and reliability of this reconstructive method.
